# Comparative Serum Analyses Identify Cytokines and Hormones Commonly Dysregulated as Well as Implicated in Promoting Osteolysis in MMP-2-Deficient Mice and Children

**DOI:** 10.3389/fphys.2020.568718

**Published:** 2020-09-25

**Authors:** Hassan Sarker, Eugenio Hardy, Ayman Haimour, Mahmoud A. Karim, Sabine Scholl-Bürgi, John A. Martignetti, Lorenzo D. Botto, Carlos Fernandez-Patron

**Affiliations:** ^1^Department of Biochemistry, Faculty of Medicine and Dentistry, University of Alberta, Edmonton, AB, Canada; ^2^Center of Molecular Immunology, Havana, Cuba; ^3^Department of Cell Biology, Faculty of Medicine and Dentistry, University of Alberta, Edmonton, AB, Canada; ^4^Clinic for Pediatrics I, Inherited Metabolic Disorders, Medical University of Innsbruck, Innsbruck, Austria; ^5^Department of Genetics and Genomic Sciences, Icahn School of Medicine at Mount Sinai, New York, NY, United States; ^6^Rudy L. Ruggles Biomedical Research Institute, Nuvance Health, Danbury, CT, United States; ^7^Department of Pediatrics, Division of Medical Genetics and Pediatrics, The University of Utah, Salt Lake City, UT, United States

**Keywords:** matrix metalloproteinase, multicentric osteolysis nodulosis and arthropathy, matrix metalloproteinase-2, cytokines, cortisol

## Abstract

Deficiency of matrix metalloproteinase 2 (MMP-2) causes a complex syndrome characterized by multicentric osteolysis, nodulosis, and arthropathy (MONA) as well as cardiac valve defects, dwarfism and hirsutism. MMP-2 deficient (*Mmp2*^–/–^) mice are a model for this rare multisystem pediatric syndrome but their phenotype remains incompletely characterized. Here, we extend the phenotypic characterization of MMP-2 deficiency by comparing the levels of cytokines and chemokines, soluble cytokine receptors, angiogenesis factors, bone development factors, apolipoproteins and hormones in mice and humans. Initial screening was performed on an 8-year-old male presenting a previously unreported deletion mutation c1294delC (Arg432fs) in the *MMP2* gene and diagnosed with MONA. Of eighty-one serum biomolecules analyzed, eleven were upregulated (>4-fold), two were downregulated (>4-fold) and sixty-eight remained unchanged, compared to unaffected controls. Specifically, Eotaxin, GM-CSF, M-CSF, GRO-α, MDC, IL-1β, IL-7, IL-12p40, MIP-1α, MIP-1β, and MIG were upregulated and epidermal growth factor (EGF) and ACTH were downregulated in this patient. Subsequent analysis of five additional MMP-2 deficient patients confirmed the upregulation in Eotaxin, IL-7, IL-12p40, and MIP-1α, and the downregulation in EGF. To establish whether these alterations are *bona fide* phenotypic traits of MMP-2 deficiency, we further studied *Mmp2*^–/–^ mice. Among 32 cytokines measured in plasma of *Mmp2*^–/–^ mice, the cytokines Eotaxin, IL-1β, MIP-1α, and MIG were commonly upregulated in mice as well as patients with MMP-2 deficiency. Moreover, bioactive cortisol (a factor that exacerbates osteoporosis) was also elevated in MMP-2 deficient mice and patients. Among the factors we have identified to be dysregulated in MMP-2 deficiency many are osteoclastogenic and could potentially contribute to bone disorder in MONA. These new molecular phenotypic traits merit being targeted in future research aimed at understanding the pathological mechanisms elicited by MMP-2 deficiency in children.

## Introduction

Matrix metalloproteinase 2 (MMP-2), also known as gelatinase A or 72 kDa type IV collagenase, is a member of a family of 25 different Zn^2+^-dependent endopeptidases that cleave a broad range of proteins including extracellular matrix proteins (such as collagens, laminin, fibrillin, and aggrecan) (Parks et al., 2004; Fernandez-Patron et al., 2016), cell surface receptors, growth factors as well as cytokines and chemokines (such as monocyte chemoattractant protein-3) (McQuibban et al., 2001, 2002; Morrison et al., 2009; Nissinen and Kahari, 2014). Both excessive and defective MMP-2 activity can be pro-inflammatory (Martignetti et al., 2001; Liu et al., 2006; Tuysuz et al., 2009; Nurmohamed et al., 2015; Fernandez-Patron et al., 2016; Peeters et al., 2017). In humans, deficiency of MMP-2 due to biallelic inactivating mutations in the *MMP2* gene causes a complex inflammatory syndrome comprising multicentric osteolysis, nodulosis, and arthropathy (MONA), (MIM# 259600–also known as Torg Syndrome or Torg-Winchester Syndrome), cardiac valve defects, dwarfism and hirsutism (Martignetti et al., 2001; Mosig et al., 2007; Castberg et al., 2013; Mosig and Martignetti, 2013; Bhavani et al., 2016; Fernandez-Patron et al., 2016).

Complete loss of MMP-2 function due to inactivating *MMP2* gene mutations is rare and thus, epidemiological and clinical data on MMP-2 deficiency in humans are limited. Nevertheless, there are reports of over 40 documented cases in the literature involving at least 20 different inactivating mutations in the *MMP2* gene (Bhavani et al., 2016). The pathogenesis of MONA in MMP-2 deficient patients is unknown with treatment of patients being mostly limited to palliative symptom management and physiotherapy. The rarity of MMP-2 deficiency in humans poses a great challenge when studying the pathological mechanisms elicited by this disorder. Limited access to the patients, who are young children receiving various medical treatments to manage symptoms, further limit the feasibility of studies and complicate the analyses and comparison among patients. Research on the pathogenesis of MMP-2 deficiency frequently uses *Mmp2*^–/–^ mice as a study model (Inoue et al., 2006; Mosig et al., 2007; Mosig and Martignetti, 2013). A thorough characterization of the commonalities and differences between MMP-2 deficient patients and *Mmp2*^–/–^ mice is requisite for translating findings in mice to humans. The molecular pathways connecting the loss of MMP-2 activity through inactivating mutations to the subsequent development of the complex disease conditions described above are yet to be elucidated.

In previous studies, phenotypic comparisons of human and murine MMP-2 deficiency have been mostly limited to clinical symptoms (including inflammation and pain in joints, a progressive decrease in bone mineral density and articular cartilage destruction), associated pathologies (such as congenital cardiac valve defects, dwarfism and hirsutism) and physical features (notably, craniofacial defects) observed in the murine and human conditions (Martignetti et al., 2001; Mosig et al., 2007; Castberg et al., 2013; Mosig and Martignetti, 2013; Bhavani et al., 2016; Fernandez-Patron et al., 2016). The current lack of knowledge of the pathways by which MMP-2 deficiency leads to the aforementioned disease phenotypes calls for studies, focused on cytokines, chemokines, angiogenesis and bone development factors, apolipoproteins and hormones, which are involved in mediating inflammation or bone remodeling and development (Zhang and An, 2007; Feng and McDonald, 2011).

In this research, we report molecular phenotypic pathways previously unknown to be commonly dysregulated in murine and human MMP-2 deficiency. Our analyses targeted cytokines, chemokines, angiogenesis and bone development factors, apolipoproteins and hormones in the circulation of MMP-2-deficient patients and *Mmp2*^–/–^ mice. Our results expose overlapping cytokine and chemokine expression profiles in *Mmp2*^–/–^ mice and the case of human MMP-2 deficiency. Among the serum components found to be dysregulated in both human and murine MMP-2 deficiency, bioactive cortisol and several pro-inflammatory cytokines involved in bone loss (Canalis and Delany, 2002; Weitzmann et al., 2002; Toraldo et al., 2003; Lee et al., 2009), were significantly elevated. These identified factors are likely to be intermediaries in the poorly understood pathological mechanisms elicited by MMP-2 deficiency. Further research is needed to establish and clarify their roles in the causation of the multisystem syndrome of pediatric MMP-2 deficiency. Although further research is needed to establish and clarify their roles in the causation of the multisystem syndrome of pediatric MMP-2 deficiency, the present results (i.e., identification of new molecular phenotypic traits associated with human and murine MMP-2 deficiency) are an essential first step to understanding the underlying biological mechanisms.

## Materials and Methods

The study was conducted in accordance with relevant guidelines and regulations of the Health Research Ethics Board (HREB) at the University of Alberta (study title: MMP-2 deficiency syndrome, study ID: MS4_Pro00068611). All animal protocols were approved by the University of Alberta animal care committee and executed in compliance with institutional guidelines issued by the Canada Council on Animal Care. Serum was collected with informed consent from the donors.

### Study Cohort

#### Human

Initial screening of serum factors was performed on an 8-year-old male MMP-2 deficient patient presenting a previously unreported inactivating deletion mutation c1294delC (Arg432fs) in *MMP2* gene and clinically diagnosed with MONA (summary of clinical assessment presented in Supplementary Figure S1). We selected this patient as a representative case for human MMP-2 deficiency/MONA in the initial serum screening analyses because of the following reasons: (i) the patient presented with all the hallmark symptoms of MONA (Supplementary Figure S1), (ii) extensive clinical analyses including clinical blood tests have been done to rule out secondary diseases which could cause the observed symptoms (Supplementary Figure S1), (iii) the patient received no pharmacological drugs which could potentially affect the serum factors analyzed (the patient was prescribed joint mobilization–a physical therapy intervention–as treatment). Due to the rarity of genetic MMP-2 deficiency and limited access to patient samples, we deemed this unconventional approach necessary for this study. We followed the initial screening with analyses of selected serum factors in five other MONA diagnosed MMP-2 deficient patients of both sexes [four females and one males–including MMP-2 deficiency cases previously described (Castberg et al., 2013; Pichler et al., 2016)] of ages between 8 and 15 years were used for subsequent analyses of serum factors. The second male patient present a homozygous mutation a c.301C > T, predicting the amino acid change p.Arg101Cys (Castberg et al., 2013). The female patients present a homozygous mutation c.1699C > T in exon 11 of the *MMP2* gene that introduces a premature stop codon (p.Arg567Ter) (Pichler et al., 2016). Sera from a total of 13 different unaffected controls, which included donors of both sexes of ages between 10 and 45 years (including unaffected siblings and parents of the MMP-2 deficient patients), were used in this study (number of controls used in each experiment is stated in the figure legends). Unaffected controls were confirmed to express MMP-2 by gelatin zymography. Human serum samples were collected, frozen, transported from the sites of collection in dry ice and stored at −80°C before subsequent analysis.

#### Mouse

Plasma (EDTA-treated) from four *Mmp2*^–/–^ to four WT male mice aged ten to 15 weeks were used for multiplex assays. Sera from eight *Mmp2*^–/–^ to six WT male mice aged 10–15 weeks were used for cortisol analyses. Mice were sacrificed and plasma were collected in the morning between 8:00 am and 11:00 am and stored at −80°C until subsequent analyses.

### Multiplex Assays to Quantify Serum Components

#### Assessment of Markers of Inflammation in *Mmp2*^–/–^ Mice

Mouse plasma and chemokine concentrations were determined using a multiplex immunoassay (Millipore MILLIPLEX; cat# MCYTMAG-70K-PX32) analyzed with a BioPlex 200 (Bio-Rad, United States). Mouse cytokines and chemokines measured include: Eotaxin, G-CSF, GM-CSF, IFN-γ, IL-1α, IL-1β, IL-2, IL-3, IL-4, IL-5, IL-6, IL-7, IL-9, IL-10, IL-12p40, IL-12p70, IL-13, IL-15, IL-17, IP-10, KC, LIF, LIX, MCP-1, M-CSF, MIG, MIP-1α, MIP-1β, MIP-2, RANTES, TNFα, and VEGF.

#### Assessment of Inflammation Markers in MMP-2 Deficient Patient

Human serum cytokine and chemokine concentrations were determined using a 42-plex (Millipore MILLIPLEX; cat# HCYTOMAG-60K) and a 48-plex (Eve Technologies, Canada, cat#HD48) multiplex immunoassays including: sCD40L, epidermal growth factor (EGF), Eotaxin, FGF-2, Flt-3 ligand, Fractalkine, G-CSF, GM-CSF, GROα, IFNα2, IFNγ, IL-1α, IL-1β, IL-1ra, IL-2, IL-3, IL-4, IL-5, IL-6, IL-7, IL-8, IL-9, IL-10, IL-12p40, IL-12p70, IL-13, IL-15, IL-17A, IL-17E/IL-25, IL-17F, IL-18, IL-22, IL-27, IP-10, MCP-1, MCP-3, M-CSF, MDC (CCL22), MIG, MIP-1α, MIP-1β, PDGF-AA, PDGF-AB/BB, RANTES, TGFα, TNFα, TNFβ, VEGF-A. Serum concentrations of a panel of human soluble cytokine receptors consisting of sCD30, sEGFR, sgp130, sIL-1RI, sIL-1RII, sIL-2Rα, sIL-4R, sIL-6R, sRAGE, sTNFRI, sTNFRII, sVEGF-R1, sVEGF-R2, and sVEGF-R3 were measured using a multiplex immunoassay (Millipore MILLIPLEX; cat# HSCRMAG32KPX14).

#### Assessment of Vascular Growth Mediators

Human angiogenesis/growth factor array (Millipore MILLIPLEX; cat# HAGP1MAG-12K) was used to measure serum levels of Angiopoietin-2, BMP-9, EGF, Endoglin, Endothelin-1, FGF-1, FGF-2, Follistatin, G-CSF, HB-EGF, HGF, IL-8, Leptin, PLGF, VEGF-A, VEGF-C, VEGF-D.

#### Assessment of Bone Metabolism Markers

Serum concentrations of factors affecting bone development–ACTH, DKK-1, FGF-23, IL-1B, IL-6, Insulin, Leptin, PTH, OC, OPG, OPN, SOST, and TNF-α–were measured using a human bone array (Eve Technologies, Canada, cat# HBN-13-36).

#### Assessment of Lipid Carrier Proteins

Concentrations of apolipoproteins APO AI, APO AII, APO B, and APO E were measured using a human apolipoprotein 4-Plex assay (Eve Technologies, Canada).

### Measuring Serum Cortisol Concentration by Enzyme Linked Immunosorbent Assay (ELISA)

We used an enzyme linked immunosorbent assay (ELISA) kit (Enzo Life Sciences, cat# ADI-900-071) to quantitate the concentrations of cortisol in mice and human sera, following manufacturer’s instructions provided with the kit.

### Western Immunoblotting to Detect Cortisol Binding Globulin

Serum CBG was detected by Western immunoblotting. Human or mouse sera were mixed with a reducing sample buffer in a 1:20 ratio (150 mM Tris–HCl, pH 6.8, 15% (w/v) SDS, 30% (v/v) Glycerol and 10% (v/v) 2-Mercaptoethanol), heated at 95°C for 10 min; 3 μL of the resultant sample mixture was analyzed by SDS/10%-PAGE. For Western immunoblotting, the proteins were transferred from the SDS-PAGE gel onto a 0.2 μm nitrocellulose membrane (Bio-Rad, United States). The membrane was then probed with a rabbit polyclonal anti-CBG antibody (Abcam, cat# ab107368), followed by horseradish-peroxidase-conjugated goat anti-rabbit secondary antibody (Bio-Rad, United States) and the Amersham ECL Western Blot detection reagents (GE Healthcare, cat# RPN2106) as per manufacturer’s instructions.

### MMP-2/MMP-9 Detection by Substrate Zymography

Gelatin zymography system: porcine skin gelatin (Sigma, cat# G8150) was copolymerized with the 10% SDS-PAGE gel (at a final gelatin concentration of 0.2% v/v). A non-reducing sample buffer (62.6 mM Tris–HCl, pH 7.4, 25% (v/v) Glycerol, 4% (w/v) SDS and 0.01% Bromophenol blue) was mixed with serum samples in a serum/buffer ratio of 1:10 (v/v). Serum proteins were separated by gel electrophoresis in a vertical gel electrophoresis apparatus (Amersham Biosciences), at 200V constant for 2 h. The gel was washed thrice with 2.5% (v/v) Triton X-100 for 20 min each time and incubated at 37°C overnight (12 h) submerged in an enzyme assay buffer (25 mM Tris–HCl pH 7.4, 150 mM NaCl and 5 mM CaCl_2_). The gel was stained with Coomassie blue and de-stained in 25% (v/v) methanol / 10% (v/v) acetic acid. MMP-2 and MMP-9 were detected as clear substrate-lysis bands contrasting against a blue background (from Coomassie staining) in the gel.

### Statistical Analysis

SigmaPlot 13 (Systat Software, San Jose, CA, United States) was used to analyze the results and plot graphs. Data are presented as mean ± standard error of mean. One way ANOVA was performed, where appropriate (indicated in the figure legends), to determine statistical significance in the difference between two groups.

## Results

### Murine and Human MMP-2 Deficiency Result in Distinct but Overlapping Expression of Pro- and Anti-inflammatory Cytokines

**FIGURE 1:**
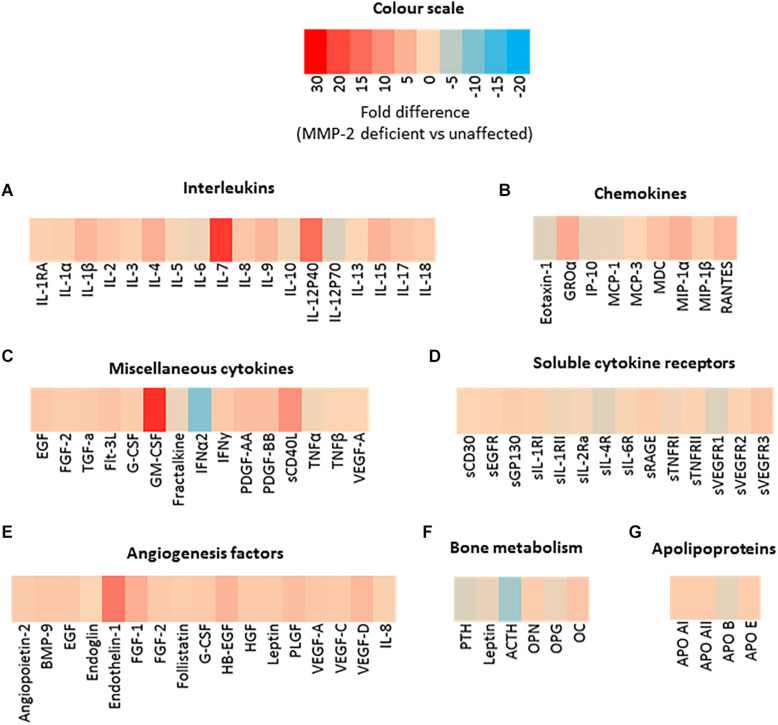
Preliminary serological screening to identify dysregulated serum factors in a case of human MMP-2 deficiency. **(A–G)** Heat map diagrams showing the relative serum levels of an array of **(A)** interleukins, **(B)** chemokines, **(A)** miscellaneous cytokines, **(D)** soluble cytokine receptors, **(E,F)** angiogenesis and bone development factors, and **(G)** apolipoproteins, in a case of human MMP-2 deficiency (8-year-old male MONA patient) compared to unaffected controls. Data are presented as fold difference between concentrations of each annotated biomolecule in the sera of MMP-2 deficient patient vs. unaffected controls (*n* = 3). Measurements were taken in duplicate (datasets presented in Supplementary Table S1).

Both murine and human MMP-2 deficiency results in severe inflammation and bone loss (Martignetti et al., 2001; Mosig et al., 2007; Mosig and Martignetti, 2013; Fernandez-Patron et al., 2016) but the MMP-2-mediated molecular pathways through which these phenotypes occur are unknown. To identify dysregulated serum factors that could be associated with the inflammatory and arthritic phenotype in MMP-2 deficiency, we first performed a targeted screen on the serum of an 8-year-old male patient with MMP-2 deficiency caused by the inactivating mutation c1294delC (Arg432fs) in the *MMP2* gene. This patient was selected as a representative case of MMP-2 deficiency for initial studies based on the presented symptoms, clinical assessment and absence of pharmacological intervention at the time of enrollment in this study. We analyzed eighty-one serum biomolecules consisting of cytokines, chemokines, angiogenesis markers, bone development factors, hormones and apolipoproteins. Compared to unaffected controls, noteworthy differences in the MMP-2 deficient patient include: (i) the cytokines GM-CSF, GRO-α, IL-12p40, IL-1β, IL-4, IL-7, IL-9, IL-15, sCD40L, and MIP-1α were substantially higher (>4-fold) (Figures 1A–C); (ii) IFN-α2 was 9-fold lower (Figure 1C); there were no notable differences in soluble cytokine receptors (Figure 1D); (iii) the vasoconstrictor Endothelin-1 (a substrate of MMP-2) (Fernandez-Patron et al., 1999) was 13-fold higher (Figure 1E); (iv) ACTH was 7-fold lower (Figure 1F); (v) finally, there were no notable differences in apolipoprotein concentrations (Figure 1G).

**FIGURE 2:**
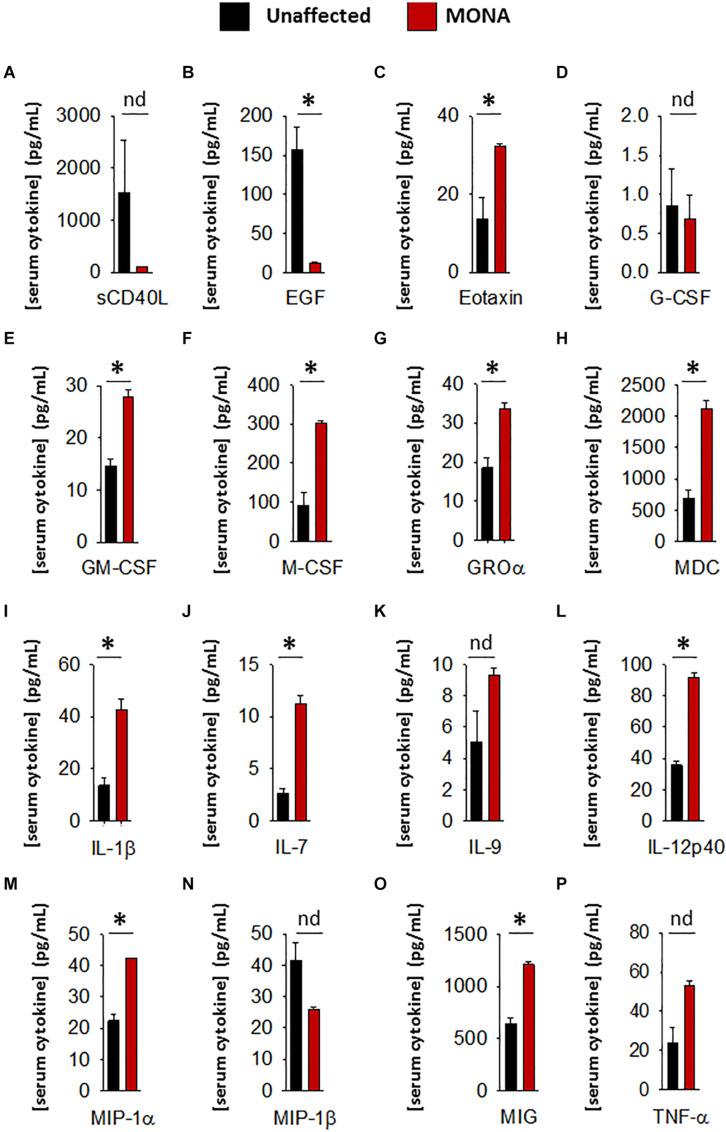
Complementary analyses of a panel of selected cytokines in the 8-years-old male MONA patient to confirm dysregulation. **(A–P)** Bar graphs comparing concentrations of cytokines and chemokines in sera of MONA patient vs. unaffected controls (*n* = 3). The panel of selected cytokines was measured using a cytokine 48-plex assay (Eve Technologies). Data are presented as mean ± SE of the mean. Measurements were taken in duplicate (datasets presented in Supplementary Table S2). *p < 0.05, comparisons MONA vs. unaffected control group (Holm–Sidak method); nd, no difference.

To increase confidence in the observed dysregulated cytokines in the initial screening, we conducted a second multiplex cytokine assay (48-plex) on the sera of the MMP-2 deficient patient along with unaffected controls. We observed a significant upregulation in Eotaxin, GM-CSF, M-CSF, GRO-α, MDC, IL-1β, IL-7, IL-12p40, MIP-1α, MIP-1β, and MIG, whereas EGF was downregulated in the patient (Figure 2). To determine whether these observations can be generalized to all the MMP-2 deficient patients in the study, we conducted confirmatory multiplex cytokine analyses on the sera of a group of five MMP-2 deficient patients diagnosed with MONA (four female and one male) compared to six unaffected controls (five female and one male). These analyses showed a significant upregulation in Eotaxin, IL-7, IL-12p40, and MIP-1α, and a downregulation in EGF in the MMP-2 deficient patients (Table 1).

**TABLE 1:** Comparison of analyses of circulating cytokine levels in human and murine MMP-2 deficiency.

Factor	Control	MONA	*p*-value	WT plasma	*Mmp2*^–/–^ plasma	*p*-value
Eotaxin	19.5 ± 4.12	55.1 ± 11.5	0.009	49.8 ± 2.72	572 ± 6.72	<0.001
G-CSF	6.84 ± 3.61	0.721 ± 0.179	0.16	0.640 ± 0.00	38.8 ± 7.89	0.04
GM-CSF	24.4 ± 6.15	16.7 ± 3.03	0.317	36.1	45.21	–
IFNy	1.48 ± 0.312	0.664 ± 0.251	0.078	ND	2.37 ± 1.68	–
IL-1a	33.3 ± 6.90	34.0 ± 8.48	0.952	10.22 ± 0.00	59.9 ± 11.1	0.046
IL-1B	31.2 ± 10.5	29.7 ± 8.04	0.91	8.01 ± 3.40	23.4 ± 0.590	0.047
IL-2	1.83 ± 0.971	0.896 ± 0.320	0.425	3.86 ± 0.930	3.65 ± 0.658	0.874
IL-3	0.055 ± 0.0156	0.311 ± 0.165	0.122	ND	ND	–
IL-4	1.30 ± 0.267	0.845 ± 0.194	0.214	ND	0.12 ± 0.035	–
IL-5	5.07 ± 2.77	3.34 ± 1.09	0.603	4.70 ± 0.124	5.72 ± 0.0884	0.022
IL-6	0.633 ± 0.135	0.706 ± 0.0900	0.679	ND	1.34 ± 0.428	–
IL-7	3.54 ± 0.745	9.37 ± 1.54	0.005	12.3 ± 1.40	12.1 ± 2.28	0.935
IL-9	3.84 ± 1.40	7.51 ± 1.54	0.111	3.46 ± 2.44	ND	–
IL-10	0.908 ± 0.319	4.60 ± 1.99	0.074	7.22 ± 4.20	3.03 ± 1.02	0.434
IL-12p40	52.4 ± 12.3	140 ± 15.1	0.001	2.78 ± 1.28	3.84 ± 1.81	0.68
IL-12p70	1.74 ± 0.591	1.82 ± 0.631	0.928	9.96 ± 0.382	5.25 ± 1.09	0.055
IL-13	48.3 ± 5.91	37.9 ± 8.16	0.318	41.1 ± 24.5	72.4 ± 18.1	0.412
IL-15	11.3 ± 1.90	17.1 ± 6.34	0.367	1.28 ± 0.00	36.8 ± 13.7	0.122
IL-17	11.4 ± 6.51	17.3 ± 8.38	0.582	ND	0.885 ± 0.237	–
IP-10	43.1 ± 11.6	37.4 ± 9.32	0.718	13.4 ± 0.711	73.1 ± 3.83	0.004
MCP-1	387 ± 41.6	320 ± 39.8	0.286	ND	ND	–
M-CSF	105 ± 25.8	186 ± 65.5	0.28	25.6 ± 11.5	13.7 ± 9.47	0.507
MIG	1060 ± 202	1650 ± 521	0.292	13.0 ± 0.979	112 ± 4.96	0.003
MIP-1a	18.8 ± 2.20	45.5 ± 9.49	0.015	65.7 ± 2.676	8.16 ± 4.01	0.007
MIP-1B	45.2 ± 5.67	31.0 ± 3.87	0.078	14.5 ± 4.43	18.1 ± 3.15	0.578
RANTES	3800 ± 1120	11700 ± 8370	0.33	11.6 ± 3.79	50.8 ± 3.92	0.019
TNFa	28.6 ± 4.05	38.0 ± 6.15	0.22	7.49 ± 4.39	1.28 ± 0.00	0.293
VEGF	271 ± 70.6	152 ± 27.2	0.18	0.155 ± 0.103	0.595 ± 0.0247	0.053

*The panel of selected human cytokines and chemokines was measured in sera from MMP-2 deficient MONA patients (*n* = 5) and unaffected controls (*n* = 6) using a cytokine 48-plex assay (Eve Technologies). The panel of selected murine cytokines and chemokines was measured in pooled plasma of *Mmp2*^–/–^ (*n* = 4) and WT (*n* = 4) mice using the cytokine 32-plex assay (Eve Technologies). *p*-values obtained from comparisons of MONA vs. unaffected control group and *Mmp2*^–/–^ (*n* = 4) vs. WT (*n* = 4) mice (Holm–Sidak method); ND, not detected. Measurements were taken in duplicate (datasets presented in Supplementary Tables S3, S4).*

**FIGURE 3:**
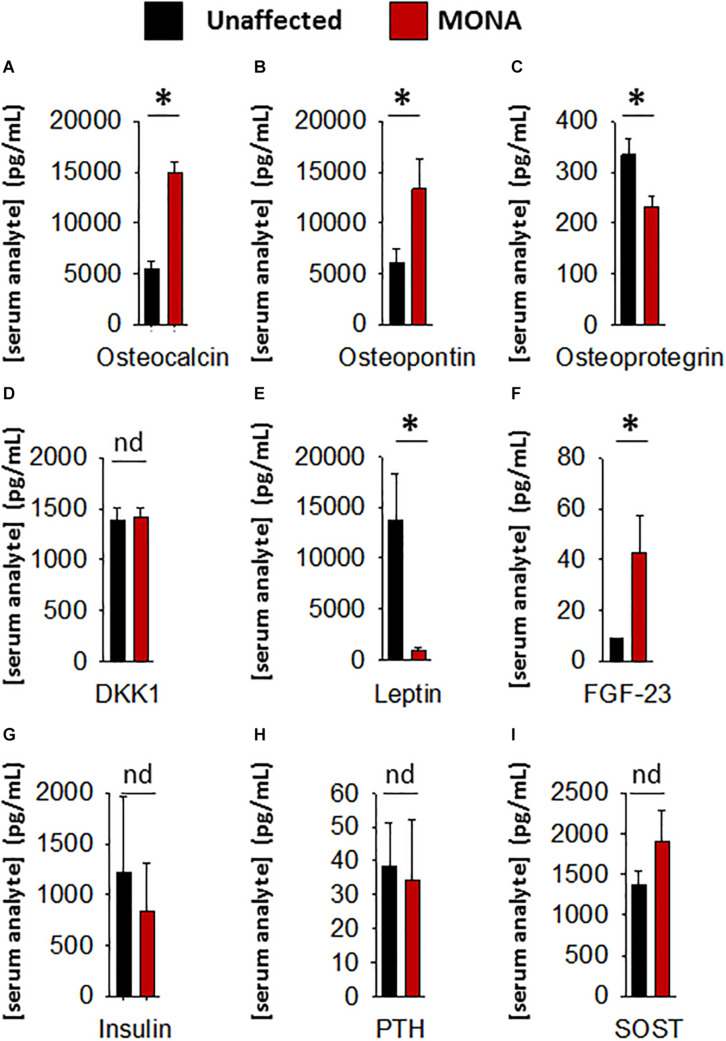
Analyses of a panel of selected bone metabolism markers in the sera of MONA patients. **(A–I)** Bar graphs comparing concentrations of bone metabolism markers in the sera of MONA patients (*n* = 5) vs. unaffected controls (*n* = 6). The panel of selected markers of bone development was measured using a 13-plex human bone array (Eve Technologies). Data are presented as mean ± SE of the mean. Measurements were taken in duplicate (datasets presented in Supplementary Table S2). *p < 0.05, comparisons MONA vs. unaffected control group (Holm–Sidak method); nd, no difference.

Further determinations of serum factors that affect bone growth and development in the same group of MMP-2 deficient patients (compared to unaffected controls) showed significant upregulation in osteocalcin, osteopontin, and FGF-23, and downregulation in osteoprotegrin and leptin, while DKK1, SOST, PTH, and insulin showed no difference (Figure 3).

*Mmp2*^–/–^ mice are the standard study model for research on the pathogenesis of MMP-2 deficiency (Inoue et al., 2006; Mosig et al., 2007; Mosig and Martignetti, 2013). Thus, a thorough characterization of the commonalities and differences between MMP-2 deficient patients and *Mmp2*^–/–^ mice is requisite for translating findings in mice to humans. Identifying molecular phenotypic traits common to murine and human MMP-2 deficiency would provide a starting point for research aimed at understanding the pathological mechanisms elicited in human MMP-2 deficiency using the murine model.

To determine whether the dysregulated cytokines observed in the case of human MMP-2 deficiency are also dysregulated in murine MMP-2 deficiency, we measured the levels of thirty-two cytokines and chemokines in the plasma of age- and sex matched MMP-2 knockout (*Mmp2*^–/–^) mice (*n* = 4) and compared them to wild type (WT) (*n* = 4). The cytokines Eotaxin, MIG, IL-1α, IL-1β, IL-5, G-CSF, RANTES, KC, IP-10, and LIX (CXCL5) were significantly upregulated, whereas the cytokine MIP-1 α and LIF were significantly downregulated in the plasma of *Mmp2*^–/–^ mice compared to WT (Table 1). There was no statistically significant difference between the two groups in M-CSF, TNF-α, IFN-γ, IL-2, IL-3, IL-4, IL-6, IL-7, IL-9, IL-10, IL-12 p40, IL-12p70, IL-13, IL-15, IL-17, MCP-1, MIP-2, and VEGF (Table 1). Together, these results reveal a distinct yet overlapping cytokine expression profiles in the circulation of MMP-2 deficient mouse and human (Figure 3A). Indeed, Eotaxin, IL-1β, and MIG were upregulated in the circulation of *Mmp2*^–/–^ mice and the MMP-2 deficient patients (Tables 1, 2). The cytokines IL-1α, G-CSF, and RANTES were uniquely upregulated in the circulation of *Mmp2*^–/–^ mice. In contrast, IL-7, IL-12 p40, GM-CSF, and M-CSF were uniquely dysregulated in the circulation of MMP-2 deficient patients (Figure 2 and Tables 1, 2).

**TABLE 2:** Summary of dysregulated cytokines in human and murine MMP-2 deficiency.

Factors	Action	Reference	MONA (serum)	*Mmp2*^–/–^ (plasma)	*Mmp2*^–/–^ (heart)
Eotaxin	Induces migration of pre-osteoclast and increases bone resorption	Kindstedt et al., 2017	up	up	up
EGF	Stimulates proliferation of osteoblast progenitor cells	Tamama et al., 2006; Zhang et al., 2011	down	–	–
IL-1α	Induces osteoclast differentiation via MITF activation	Kim et al., 2009	nd	up	nd
IL-1β	Induces RANKL expression and osteoclast differentiation	Jules et al., 2012; Ruscitti et al., 2015	up	up	nd
IL-7	Stimulates osteoclast formation by inducing RANKL and M-CSF expression and also via STAT5 activation	Weitzmann et al., 2000; Toraldo et al., 2003; Kim et al., 2017	up	nd	up
IL-12p40	Subunit of IL-23; promotes osteoclastogenesis via inducing RANKL and RANK expression	Chen et al., 2008; Kang and Zhang, 2014	up	nd	up
G-CSF	Increases osteoclast bone-resorption activity	Hirbe et al., 2007	nd	up	up
GM-CSF	Increases formation of multinuclear bone-resorbing osteoclasts	Lee et al., 2009; Ruef et al., 2017	up	nd	nd
M-CSF	Induces osteoclast differentiation, survival, proliferation, and maturation	Ruef et al., 2017	up	nd	nd
MIP-1α	Promotes migration and activation of osteoclasts	Watanabe et al., 2004	up	down	up
RANTES	Promotes cell migration and survival of osteoblasts	Yano et al., 2005	nd	up	up
MIG	Stimulates osteoclast migration and adhesion	Kwak et al., 2005	up	up	up
OPG	Decoy receptor of RANKL; inhibits osteoclast differentiation	Bucay et al., 1998; Takayanagi, 2007; Mori et al., 2013	down	–	–

*Upregulation or downregulation of cytokines in sera of MONA patients vs. controls (Figures 1–3, Table 1, and Supplementary Tables S1, S2, S4), plasma of *Mmp2*^–/–^ vs. WT (Table 1 and Supplementary Table S3) and heart tissue of *Mmp2*^–/–^ vs. WT (Supplementary Table S3; Berry et al., 2015); nd: no difference.*

Together, our results identify cytokines dysregulated in human and murine MMP-2 deficiency that are implicated in bone loss (Kitas et al., 1988; Canalis, 1996; Suda et al., 1999; Weitzmann et al., 2002; Toraldo et al., 2003; Takayanagi, 2007; Lee et al., 2009; Rauber et al., 2017).

### MMP-2 Deficiency Is Associated With Increased Circulatory Levels of Cortisol and Decreased Cortisol Binding Globulin

**FIGURE 4:**
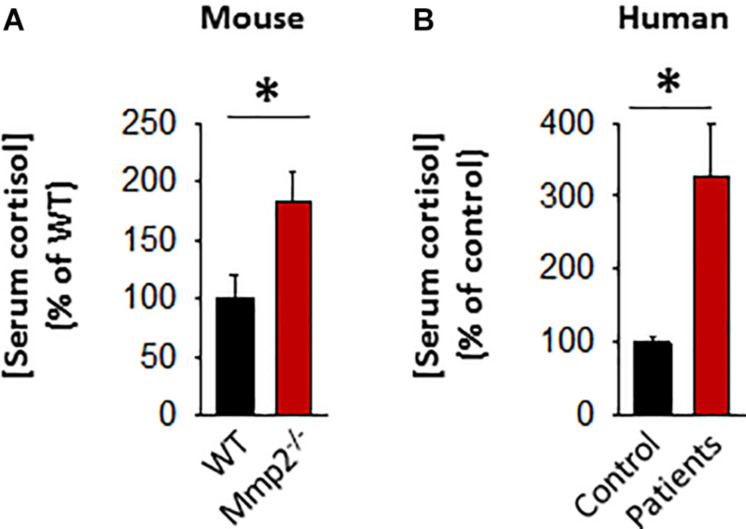
**(A)** Bar graph showing serum cortisol concentration in *Mmp2*^– / –^ mice (*n* = 6) compared to WT mice (*n* = 6). **(B)** Bar graph showing serum cortisol concentration in MMP-2 deficient humans (*n* = 6) compared to unaffected controls (*n* = 8). *p < 0.05, comparison of *Mmp2*^– / –^ vs. WT one-way ANOVA. Duplicate readings of standards taken to ensure technical reproducibility (datasets presented in Supplementary Table S5).

The initial serum screening of the 8-year-old male MMP-2 deficient patient showed relatively lower levels of serum adrenocorticotropic hormone (ACTH) in MMP-2 deficient serum compared to unaffected controls (Figure 1F). This raised the question whether circulating cortisol levels is dysregulated in the MMP-2 deficient patient and if so, can this dysregulation be generalized to other MMP-2 deficient patients and mice? To answer this question, we measured serum cortisol levels in MMP-2 deficient patients as well as *Mmp2*^–/–^ mice along with their respective unaffected controls by ELISA. The results showed that serum cortisol concentrations were significantly higher in both MMP-2 deficient humans and *Mmp2*^–/–^ mice (Figures 4A,B) compared to their respective unaffected controls.

Approximately up to 90% of cortisol in circulation is bound to the cortisol binding protein (CBG) which keeps cortisol biologically inactive (Siiteri et al., 1982). Hence, we measured serum CBG protein levels in MMP-2 deficient patients and mice to determine whether CBG is also elevated in response to cortisol elevation in MMP-2 deficiency to suppress its biological activity. The results showed that there is no difference in CBG levels in MMP-2 deficient patients tested compared to unaffected controls (Supplementary Figures S2A,B), whereas *Mmp2*^–/–^ mice had significantly lower serum CBG compared to wild type (Supplementary Figures S2C,D). The results indicate that there is no compensatory increase in CBG in response to elevated cortisol to suppress its biological activity and hence overall biologically active cortisol is elevated in the sera of both MMP-2 deficient mice and humans.

## Discussion

In this research, we aimed to advance the phenotypic characterization of MMP-2 deficiency by identifying molecular traits that are similarly dysregulated in MMP-2 deficient patients and *Mmp2*^–/–^ mice which are the standard study model for the rare multisystem pediatric syndrome elicited by MMP-2 deficiency in humans. Due to the rarity of genetic MMP-2 deficiency and limited access to patient samples, we adopted an approach where we selected an 8-year old male MMP-2 deficient MONA patient as a representative case for human MMP-2 deficiency/MONA in the initial serum screening analyses. Our selection of this patient was based on the following: (i) the patient presented with all the hallmark symptoms of MONA (Supplementary Figure S1), (ii) extensive clinical analyses including clinical blood tests have been done to rule out secondary diseases which could cause the observed symptoms (Supplementary Figure S1), (iii) the patient received no pharmacological drugs which could potentially affect the serum factors analyzed (the patient was prescribed joint mobilization–a physical therapy intervention–as treatment). MONA patients receive treatment with pharmacological drugs (such as bisphosphonates to reduce skeletal pain and increase bone mineral density (Pichler et al., 2016) which may have unpredictable side effects and affect the upregulation or downregulation of the serum factors analyzed. Therefore, it was necessary to adopt this unconventional approach of finding a representative case of human MMP-2 deficiency where interfering factors such as pharmacological drugs and secondary diseases could be ruled out. We validated our findings from analyses of the representative case with *Mmp2*^–/–^ mice and a cohort of five MMP-2 deficient patients and found serum components that are similarly dysregulated in MMP-2 deficient mice and patients.

### The Cytokines Dysregulated in MMP-2 Deficiency Include Known Inducers of Osteoporosis

The dynamic bone remodeling process is maintained by a balance and signaling between bone-forming osteoblasts (which produce bone matrix proteins) (Blair et al., 2017) and bone-resorbing multinucleated osteoclasts (Suda et al., 1999; Lee et al., 2009). Chronic inflammation, as present in MMP-2 deficiency (Martignetti et al., 2001; Mosig et al., 2007; Tuysuz et al., 2009; Castberg et al., 2013; Mosig and Martignetti, 2013; Bhavani et al., 2016; Fernandez-Patron et al., 2016), alters the balance between osteoclasts and osteoblasts favoring more bone resorption which then leads to osteoporosis (Hardy and Cooper, 2009).

Among the panel of selected markers of bone metabolism analyzed, FGF-23 was upregulated in MONA patients. Among the regulators of bone turnover, osteoprotegrin (OPG) was upregulated, whereas dickkopf-1 (DKK-1) and sclerostin (SOST) were unchanged in MONA patients. Although, further research is necessary to establish the consequences of the observed upregulation in FGF-23 and OPG in MMP-2 deficiency, these two markers could potentially contribute to exacerbating osteolysis in the patients. FGF-23 is mainly secreted by osteocytes and osteoblasts and is upregulated in the circulation in cases of bone disorders characterized by softening and weakening of bones (low bone mineral density) including all types of hypophosphatemic rickets (Guo and Yuan, 2015). FGF-23 inhibits bone mineralization (Wang et al., 2008), but the mechanism of action remains unclear. OPG acts as a decoy receptor for RANKL and inhibit osteoclastogenesis, and a downregulation in OPG (observed in MONA patients) is associated with increased osteoporosis (Bucay et al., 1998; Takayanagi, 2007; Mori et al., 2013).

Among the pro-inflammatory cytokines that were elevated in the circulation of *Mmp2*^–/–^ mice and/or MMP-2 deficient patients, IL-1α, IL-1β, IL-7, IL-12p40, G-CSF, GM-CSF, M-CSF, MIP-1α, and MIG are implicated in the promotion of bone resorption by stimulating osteoclast migration, proliferation, differentiation or activation (Table 2; Suda et al., 1999; Weitzmann et al., 2000; Canalis and Delany, 2002; Watanabe et al., 2004; Kwak et al., 2005; Hirbe et al., 2007; Takayanagi, 2007; Chen et al., 2008; Kim et al., 2009; Lee et al., 2009; Zhang et al., 2011; Jules et al., 2012; Kang and Zhang, 2014; Yokota et al., 2014; Ruscitti et al., 2015; Kindstedt et al., 2017). Although further research is necessary to establish the consequences of the upregulation in these cytokines in the pathological mechanisms elicited in MMP-2 deficiency, a brief synthesis based on evidence from the literature on how these cytokines could contribute to increased osteolysis in MMP-2 deficiency are as follows. Osteoclast maturation is regulated by factors such as the receptor activator of NF−κB ligand and M-CSF, in addition to TNF-α and GM-CSF (Suda et al., 1999; Takayanagi, 2007). IL-7, which was commonly elevated in the circulation of MONA patients and heart of *Mmp2*^–/–^ mice (Table 2), stimulates osteoclast formation by up-regulating the T-cell production of soluble osteoclastogenic cytokines RANKL and M-CSF (Weitzmann et al., 2000). Moreover, *in vivo* administration of IL-7 induces bone loss as well as blocks new bone formation in mice (Weitzmann et al., 2002; Toraldo et al., 2003). IL-12p40 was consistently elevated across all MONA patients (Figures 1, 2 and Table 1) and also upregulated in the heart of *Mmp2*^–/–^ mice (Berry et al., 2015). IL-12p40 as a subunit of IL-23 is associated with osteolysis in mice (Xu et al., 2020) and functions to promote osteoclastogenesis by inducing RANKL and RANK expression (Chen et al., 2008; Kang and Zhang, 2014). The cytokine GM-CSF, elevated in the 8-year-old MMP-2 deficient patient, induces the fusion of mononuclear osteoclasts to bone-resorbing multinuclear osteoclasts in mice (Lee et al., 2009). High levels of GM-CSF and M-CSF in systemic inflammatory diseases are suggested to increase osteoclast progenitor cell pools (in the bones, bone marrow and blood) which upon homing to the bones can lead to an increase in the number of osteoclasts and bone resorption (Ruef et al., 2017; Figure 3C). GM-CSF is a promising therapeutic target in the treatment of rheumatoid arthritis–an autoimmune disease characterized by joint inflammation which can lead to local and systemic osteoporosis (Goldring and Gravallese, 2000; Avci et al., 2016; Guo et al., 2019). Mavrilimumab, a monoclonal antibody against GM-CSF receptor, has demonstrated promising results in the treatment of rheumatoid arthritis in clinical trials and is being considered as an additional therapeutic option for rheumatoid arthritis patients who are resistant to the available targeted drugs (Avci et al., 2016; Burmester et al., 2017, 2018; Crotti et al., 2019). Further research is necessary to establish the specific consequences of GM-CSF in MMP-2 deficiency, and to test the possibility of targeting GM-CSF to manage inflammation and prevent bone loss in MONA.

**FIGURE 5:**
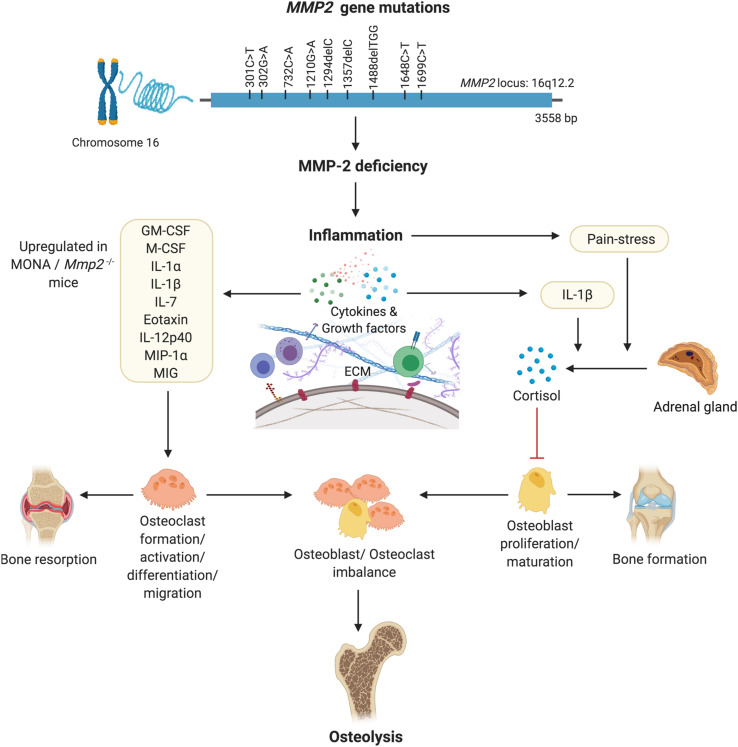
A hypothesized pathway of MMP-2 deficiency mediated osteoporosis. Denaturing mutations in the *MMP2* gene leading to MMP-2 deficiency mediated inflammation results in increased circulating levels of pro-inflammatory cytokines known to increase the formation, migration, differentiation and activation of osteoclasts. Pain-stress resulting from inflammation induces an increase in secretion of cortisol which has an inhibitory effect on osteoblast proliferation and maturation. Consequently, an imbalance in the population of bone forming osteoblasts and bone-resorbing osteoclasts results in osteolysis. Figure made using BioRender.

The variations in the dysregulated serum cytokines observed across MONA patients (Figures 1, 2 and Table 1; 8-year-old male patient vs. all five MONA patients) likely arise due to different medications and treatments the patients receive in to manage pain and inflammation among other uncontrollable factors. Three of these patients receive bisphosphonates to reduce skeletal pain and increase bone mineral density (Pichler et al., 2016). Despite the existing variations in treatments between the patients, the cytokines Eotaxin, IL-7, IL-12p40, and MIP-1α were upregulated, and EGF was downregulated across all patients (Figure 2 and Table 1). Although the consequences of these changes specifically in MMP-2 deficiency need to be established, the cytokines Eotaxin, IL-7, IL-12p40, and MIP-1α could contribute to increased osteolysis in MMP-2 deficiency through their known osteoclastogenic functions causing increased bone resorption (Table 2 and Figure 5). Also, an insufficiency of EGF in MONA could downregulate EGF’s normal function of stimulating proliferation of bone marrow stromal stem cells (the progenitor cells for osteoblasts) (Tamama et al., 2006).

An unexpected finding of this research is that only a subset of circulating pro- and/or anti-inflammatory cytokines analyzed (25% in human and 38% in mice) were dysregulated (while the majority were unchanged) in MMP-2 deficiency. The circulating factors identified here are likely to be a specific phenotypic trait of MMP-2 deficiency and could be potential targets for therapeutic intervention–an avenue for further research.

### Glucocorticoid-Induced Osteoporosis May Be a Newly Identified Phenotype Characteristic of MMP-2 Deficiency

The initial serum screening of the case of human MMP-2 deficiency showing relatively low levels of serum ACTH prompted us to look at cortisol levels in MMP-2 deficient patients. ACTH is a tropic hormone produced in the anterior pituitary gland and stimulates the release of steroid hormone cortisol from the adrenal gland (Gjerstad et al., 2018). Interestingly, our data showed the presence of significantly higher levels of cortisol in MMP-2 deficient patients compared to unaffected controls. Low ACTH typically results in low cortisol being released from the adrenal gland, however presence of low ACTH could also imply presence of high serum cortisol which lowers ACTH release by a negative feedback mechanism (Gjerstad et al., 2018).

Age, sex, pharmacological interventions, and procedure and time of blood collection (Ljubijankic et al., 2008; Roelfsema et al., 2017), may potentially affect serum cortisol levels in the patients. However, these factors are challenging to account for when studying a rare pediatric condition such as MMP-2 deficiency because of the limited number of diagnosed patients in the world. Furthermore, there are practical difficulties in accessing to these patients and the various medical treatments prescribed to manage their inflammation and pain are likely to alter the expression of many cytokines.

In humans, circulating cortisol levels are higher on average during the day compared to night (Krieger et al., 1971; Weitzman et al., 1971), whereas in mice circulating cortisol levels are higher during night (Chung et al., 2016). For practical reasons, human sera collection in the clinic typically occurs during the day (when patients and healthy controls visit the hospital and follows strict protocol). The animal model of MMP-2 deficiency, *Mmp2*^–/–^ mice, allowed us flexibility in terms of controlling the aforementioned variables that may affect circulating cortisol. Yet, unaware of a possible influence of MMP-2 levels on cortisol, we collected mice sera in the morning between the hours of 8:00 am and 11:00 am, a period of the day when circulating cortisol levels in mice are the lowest (Gong et al., 2015). Interestingly, we found that serum cortisol levels were significantly higher whereas CBG levels were significantly lower in *Mmp2*^–/–^ mice, compared to WT mice. Our findings are in agreement with previous research (Yang et al., 2002), which had reported a strong association between lower levels of MMP-2 protein in circulation with higher levels of plasma cortisol in humans. Thus, our data suggest a strong association between MMP-2 deficiency and increased levels of circulating cortisol and decreased levels of the endogenous cortisol inhibitor CBG.

Matrix metalloproteinase 2 deficient patients and *Mmp2*^–/–^ mice present with chronic inflammation of the joints, and as such, upon movement, they experience chronic pain (Mosig et al., 2007; Bhavani et al., 2016)–a stressor capable of inducing the stress response and intensifying cortisol secretion which has acute anti-inflammatory effects (Hannibal and Bishop, 2014). The chronic nature of the pain-related stressor could cause chronic reactivation of the stress response and repeated surges in cortisol secretion (Hannibal and Bishop, 2014). Thus, chronic pain-related stress may have resulted in the elevated serum cortisol levels observed in MMP-2 deficient patients and *Mmp2*^–/–^ mice. Further, IL-1β was upregulated in both human and murine MMP-2 deficiency (Figures 1, 2 and Tables 1, 2). While the specific effect of IL-1β upregulation in MMP-2 deficiency need to be established, a likely effect of IL-1β upregulation is to directly stimulate the release of cortisol from the adrenal glands (Figure 5; Besedovsky and del Rey, 1996; Ebrahimi et al., 2019).

While our observations associate increased circulating bioactive cortisol with MMP-2 deficiency, further studies are necessary to elucidate the molecular mechanisms of this association and the specific consequences of elevated cortisol in the pathogenesis of MONA. Conceivably, the potential consequences of increased cortisol in the MMP-2 deficiency are likely to be through the known mechanisms by which cortisol affects bone metabolism. Glucocorticoids including cortisol inhibit calcium absorption, an increase of urinary calcium excretion (Canalis and Delany, 2002), inhibit bone formation as well as increase bone resorption, which lead to osteoporosis after continued exposure to elevated cortisol (Hahn et al., 1979; Canalis, 1996; Dalle Carbonare et al., 2001). Cortisol increases the expression of the crucial osteoclastogenesis factor M-CSF (Rubin et al., 1998) while decreasing the production of the anti-resorptive RANK-L decoy receptor osteoprotegrin (downregulated in MONA patients; Figure 3C; Hofbauer et al., 1999), therefore promoting bone resorption and osteoclastogenesis while disrupting the coupled osteoblast-osteoclast interaction (Canalis and Delany, 2002). Cortisol can decrease osteoblasts replication and slow their differentiation into mature osteoblasts (Pereira et al., 2001; Canalis and Delany, 2002). In addition, cortisol inhibits the synthesis of type 1 collagen, the most abundant bone matrix protein and a major constituent in bone mineralization (Tzaphlidou, 2008), by transcriptional and post-transcriptional mechanisms in osteoblasts (Delany et al., 1995a). Further, cortisol increases the half-life of collagenase type III (MMP-13) transcripts in osteoblasts, resulting in increased matrix collagen degradation (Delany et al., 1995b). Thus, further research is needed to establish cortisol as a target for therapeutic intervention in reducing osteoporosis in MMP-2 deficiency.

## Conclusion

In conclusion, this study has identified new molecular phenotypic traits, common to murine and human MMP-2 deficiency, which merit being targeted in future research aimed at understanding the multisystem pathologies elicited by MMP-2 deficiency in children.

## Data Availability Statement

The raw data supporting the conclusions of this article will be made available by the authors, without undue reservation.

## Ethics Statement

The studies involving human participants were reviewed and approved by Health Research Ethics Board–Biomedical Panel. Written informed consent to participate in this study was provided by the participants’ legal guardian/next of kin. The animal study was reviewed and approved by Health Research Ethics Board, University of Alberta.

## Author Contributions

HS and CF-P conceived the study, designed the experiments, and wrote the manuscript. HS and MK conducted the experiments. AH and EH assisted in literature research, the experimental design, and writing the manuscript. LB, JM, and SS-B recruited the patients and provided clinical data. All authors participated in the data analysis, performed critical revisions of the manuscript, and approved the final version of the manuscript.

## Conflict of Interest

JM was employed by company, the Rudy L. Ruggles Biomedical Research Institute, Nuvance Health, Danbury, Connecticut. The remaining authors declare that the research was conducted in the absence of any commercial or financial relationships that could be construed as a potential conflict of interest.
