# Supplementary material for: Comparative Serum Analyses Identify Cytokines and Hormones Commonly Dysregulated as Well as Implicated in Promoting Osteolysis in MMP-2-Deficient Mice and Children
Source: Front Physiol. 2020 Sep 25;11:568718. doi: 10.3389/fphys.2020.568718 (PMC7546215; doi:10.3389/fphys.2020.568718)
Supplement: Supplementary file 6 [file Data_Sheet_1.docx]

**Comparative serum analyses identify cytokines and hormones commonly dysregulated as well as implicated in promoting osteolysis in MMP-2-deficient mice and children**

**Authors:**

Hassan Sarker (1), Eugenio Hardy (2), Ayman Haimour (1), Mahmoud A. Karim (3), Sabine Scholl-Bürgi (4), John A. Martignetti (5), Lorenzo D. Botto (6), Carlos Fernandez-Patron (1*)

**Authors’ affiliations:**

(1): Department of Biochemistry, Faculty of Medicine and Dentistry, University of Alberta, Edmonton, AB T6G 2H7, Canada.

(2): Center of Molecular Immunology, P.O. Box 16040, Havana, Cuba.

(3): Department of Cell Biology, Faculty of Medicine and Dentistry, University of Alberta, Edmonton, AB T6G 2H7, Canada.

(4): Clinic for Pediatrics I, Inherited Metabolic Disorders, Medical University of Innsbruck, Anichstrasse 35, A-6020 Innsbruck, Austria.

(5) Departments of Genetics and Genomic Sciences, Icahn School of Medicine at Mount Sinai, New York, NY and Rudy L. Ruggles Biomedical Research Institute, Nuvance Health, Danbury, Connecticut.

(6): Departments of Pediatrics, Division of Medical Genetics and Pediatrics, University of Utah, Salt Lake City, UT 84108, USA.

**(*): Correspondence:**

Carlos Fernandez-Patron

Email: cf2@ualberta.ca

Department of Biochemistry

Faculty of Medicine and Dentistry

University of Alberta

Edmonton, AB T6G 2H7, Canada

**Running title:** Novel phenotypic traits of MMP-2 deficiency

**Key words:** Matrix metalloproteinase, MMP-2 deficiency, bone disorder, MONA

**
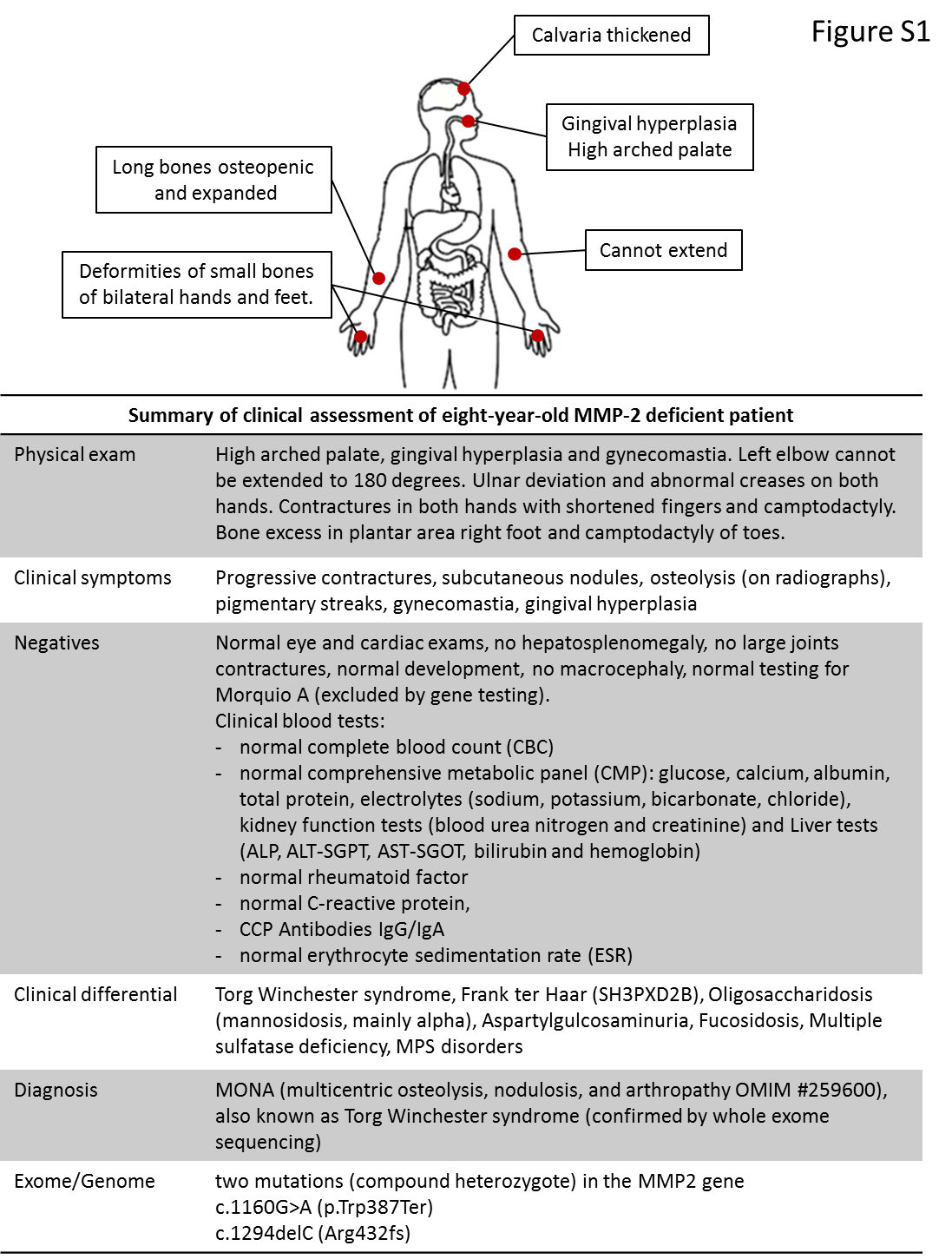
**

**Figure S1**: Summary of clinical assessment of the eight-year-old MMP-2 deficient patient.


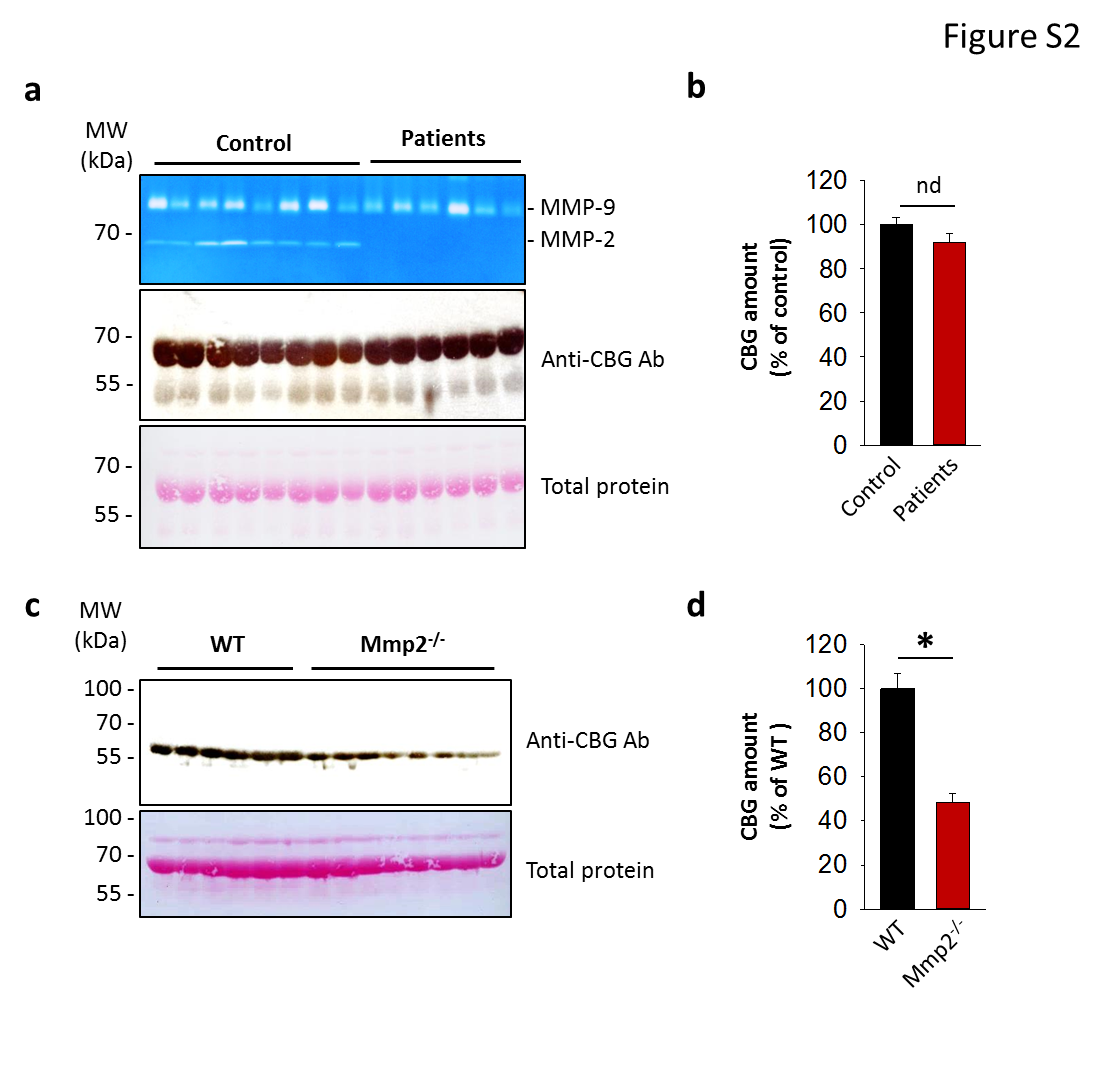
**Figure S2**: Serum cortisol binding protein in MMP-2 deficient patients, whereas cortisol binding protein is downregulated in MMP-2 deficiency.

1. Quantitation of serum cortisol binding protein in MMP-2 deficient humans and unaffected controls by western blot. Top: Gelatin zymogram of sera from MMP-2 deficient humans (n=6) and unaffected controls (n=8). Middle: Immunoblot probed with anti-CBG antibody to detect CBG in sera from MMP-2 deficient humans (n=6) and unaffected controls (n=8). Bottom: Membrane stained with ponceau red to determine total protein content. Blots shown are representative images of duplicate analyses.
2. Bar chart showing quantitation of band intensities of the blot in (a) by densitometric scanning. Band intensities of the blot probed with anti-CBG were divided by their corresponding total protein bands to normalize. *p<0.05 determined by one way ANOVA.
3. Quantitation of serum cortisol binding protein in *Mmp2^-/-^* and WT mice by western blot. Top: Immunoblot probed with anti-CBG antibody to detect CBG in sera from WT mice (n=6) and *Mmp2^-/-^* mice (n=8). Bottom: Nitrocellulose membrane stained with ponceau red to determine total protein content.
4. Bar chart showing quantitation of band intensities of the blot in (e) by densitometric scanning. Band intensities of the blot probed with anti-CBG were divided by their corresponding total protein bands to normalize. *p<0.05 determined by one way ANOVA.

**Supplementary Table S1**: Datasets generated using multiplex assays to determine serum concentrations of biomolecules presented in **Fig. 1 and Table 2**.

**Supplementary Table S2**: Datasets generated using multiplex assays to determine serum concentrations of biomolecules presented in **Fig. 2, Fig. 3** and **Table 2**.

**Supplementary Table S3**: Datasets generated using multiplex assays to determine serum concentrations of biomolecules presented in **Table 1** and **Table 2**.

**Supplementary Table S4**: Datasets generated using multiplex assays to determine serum concentrations of biomolecules presented in **Table 1 and Table 2**.

**Supplementary Table S5**: Datasets generated using ELISA to determine serum concentrations of cortisol presented in **Fig. 4**.
